# Physical Activity and Incident Pancreatic Cancer: Results From the UK Biobank Prospective Cohort

**DOI:** 10.7759/cureus.78040

**Published:** 2025-01-26

**Authors:** Borna A Assarian, Christopher D Byrne, Declan McDonnell, Zaed Hamady

**Affiliations:** 1 Human Development and Health, Faculty of Medicine University of Southampton, University Hospital Southampton NHS Foundation Trust, Southampton, GBR; 2 Hepato-Pancreato-Biliary (HPB) Unit, University Hospital Southampton NHS Foundation Trust, Southampton, GBR

**Keywords:** cancer risk, exercise, incident cancer, pancreatic cancer, pancreatic ductal adenocarcinoma, physical activity

## Abstract

Background

The relationship between physical activity and incident pancreatic cancer is poorly defined, and the evidence to date is inconsistent, largely due to small sample sizes and insufficient incident outcomes. Using the UK Biobank cohort dataset, the association between physical activity levels at recruitment and incident pancreatic ductal adenocarcinoma (PDAC) at follow-up was analysed.

Method

Physical activity, the key exposure, was quantified using Metabolic Equivalent Task (MET) values and categorised into walking, moderate, and vigorous activity. These categories were each analysed in quartiles. Summed activity was analysed both in quartiles and using International Physical Activity Questionnaire (IPAQ) activity levels (low, moderate, high). Univariate hazard ratios (HRs) and multivariable-adjusted HRs (aHRs) with 95% confidence intervals (CIs) were calculated using Cox regression analyses.

Results

A total of 542 incident PDAC cases and 2,139 controls (1:4 matching for age and sex) with a median (IQR) follow-up of 6.8 (1.7) years were analysed. No significant association was found in walking, moderate, and vigorous activities. In summed activity, only the 3rd quartile showed a statistically significant inverse association with PDAC risk (aHR 0.67, 95% CI 0.52-0.86, p<0.01). For IPAQ activity, the moderate and high activity groups showed borderline statistically significant associations with incident PDAC (aHR 0.80, 95% CI 0.63-1.00, p=0.05, and aHR 0.80, 95% CI 0.64-1.01, p=0.06, respectively).

Conclusion

The large UK Biobank cohort study did not show a strong association between physical activity levels and the development of incident PDAC.

## Introduction

Background

Pancreatic cancer is the 12th most common cancer worldwide, with approximately 500,000 cases per year [1]. It is ranked as the cancer with the seventh-highest mortality rate and leads to approximately 470,000 deaths each year, which is almost equal to the number of incident cases [1]. This cancer has a survival rate of less than 10%, the lowest of all common cancers [2-4]. It has also been projected that pancreatic cancer will surpass breast cancer to become the third leading cause of cancer death in Europe [5-7]. The incidence and mortality of pancreatic cancer are both on the rise [8]. In most cases, pancreatic cancer symptoms do not present in the earlier stages, and there are no effective screening methods [9]. This leads to later diagnoses, which are clinically more challenging to manage. This highlights the importance of advancements in identifying preventable risk factors for this deadly cancer.

Physical activity has known inverse associations with different cancers, including colorectal, breast, and endometrial cancers [10-12]. However, the literature is inconclusive about the association between physical activity and pancreatic cancer.

Aetiology and risk factors

Pancreatic cancer can be divided into two categories: exocrine and neuroendocrine. Exocrine pancreatic cancers arise from exocrine cells in the pancreas, which secrete enzymes to aid digestion. Pancreatic ductal adenocarcinoma (PDAC) is a type of exocrine cancer and accounts for about 95% of pancreatic cancers [4]. Neuroendocrine pancreatic cancers arise from endocrine cells involved in secreting hormones.

The exact aetiology of pancreatic cancer is unknown. It is likely to involve an interaction between environmental and genetic factors. Risk factors for this cancer include increasing age [2], affected first-degree relatives [13], smoking [14], diabetes [8,15-17], alcohol [2], obesity [2], chronic pancreatitis [18,19], and hereditary pancreatitis [18,19]. Furthermore, the genetic aetiology of pancreatic cancer likely involves mutations that are acquired or inherited, such as the mutated K-ras gene [20,21]. There is growing evidence of other potential risk factors for pancreatic cancer; however, there remains uncertainty about whether physical activity affects the risk of pancreatic cancer [22].

Physical activity has been shown to reduce body fat, improve insulin sensitivity, and reduce chronic inflammation [23-26]. These factors have been linked with pancreatic cancer development and are possible biological mechanisms by which physical activity could reduce pancreatic cancer incidence.

A number of studies have investigated the association between physical activity and pancreatic cancer incidence. These include cohort studies [27-38], case-control studies [39-42], and systematic reviews [43-46]. These studies analysed physical activity in different ways. Some studies analysed total physical activity levels, while others divided it into leisure and occupational activity. Results from the cohort and case-control studies were largely divided and inconsistent. Around half of the studies found some significant inverse associations for a subgroup of activity, which varied in type and intensity depending on the study [27,28,37-42]. Other studies showed no association between physical activity and pancreatic cancer [29-36]. Similarly, the systematic reviews also had inconsistent results [43-46].

The current evidence shows that the relationship between physical activity and pancreatic cancer incidence is unclear. Pancreatic cancer is not common, and previous studies have lacked power, with insufficient numbers of incident cases. Currently, there is a lack of cohort studies involving large populations with long-duration follow-up and sufficient numbers of incident PDAC outcomes to determine whether there is an association between physical activity and incident pancreatic cancer.

The aim of this study was to analyse the association between total physical activity and incident PDAC using the UK Biobank Study. This work was previously presented as a meeting poster abstract at the 14th Congress of the European-African Hepato-Pancreato-Biliary Association (E-AHPBA), September 15-17, 2021, in Bilbao.

## Materials and methods

Study participants and ethics

The UK Biobank is a large prospective study that aims to investigate the lifestyle, environmental, and genetic causes of a range of diseases [47]. Around 9.2 million invitations were mailed to people registered with the National Health Service (NHS), aged 40-70 years, and living within approximately 25 miles of UK Biobank assessment centres. This resulted in 502,656 participants (229,182 men and 273,474 women) being recruited between April 2007 and July 2010. Further details on the UK Biobank design and rationale are provided in the study protocol [48].

The UK Biobank has approval from the North West Multi-centre Research Ethics Committee (covers the UK), the National Information Governance Board for Health & Social Care (in England and Wales), and the Community Health Index Advisory Group (in Scotland) [49]. Informed consent was obtained from all participants [49]. The ethics reference number is 06/MRE08/65.

Assessment of exposure

At baseline, participant physical measurements were recorded, including hip and waist circumferences, height, and weight [48]. Data on socio-demographics, including age, sex, and Townsend deprivation index scores (derived from postcodes) [50], lifestyle (including alcohol intake frequency and smoking status), medical history, and other potentially health-related information were collected via a self-completed touchscreen questionnaire [48]. In the UK Biobank, the physical activity questions were derived from the International Physical Activity Questionnaire (IPAQ) short form, a validated survey instrument [51]. These questions assess activity undertaken across a comprehensive set of domains, including domestic and gardening (yard), transport-related, leisure time, and work-related activities [52].

At recruitment, participants self-reported their frequency (days per week) and duration (minutes per day) for three levels of activity: walking, moderate-intensity, and vigorous-intensity (the questions can be found in Table 1 and on the UK Biobank website) [52,53]. Participants were asked for the number of days in a typical week in which they performed at least 10 minutes of each activity category. When an answer of at least one day was given, they were additionally asked how long they usually spent performing that activity. Following IPAQ guidelines [51,52], activity recorded for less than 10 minutes a day was not counted, and durations longer than three hours were truncated to three hours.

**Table 1:** UK Biobank physical activity questions from the questionnaire

Question	Answer options
In a typical week, on how many days did you walk for at least 10 minutes at a time? (Include walking that you do at work, travelling to and from work, and for sport or leisure)	Number of days, “do not know”, “unable to walk” or “prefer not to answer”
How many minutes did you usually spend walking on a typical day?	Number of minutes, “do not know” or “prefer not to answer”
In a typical week, on how many days did you do 10 minutes or more of moderate physical activities like carrying light loads, cycling at normal pace? (Do not include walking)	Number of days, “do not know” or “prefer not to answer”
How many minutes did you usually spend doing moderate activities on a typical day?	Number of minutes, “do not know” or “prefer not to answer”
In a typical week, how many days did you do 10 minutes or more of vigorous physical activity? (These are activities that make you sweat or breathe hard such as fast cycling, aerobics, heavy lifting)	Number of days, “do not know” or “prefer not to answer”
How many minutes did you usually spend doing vigorous activities on a typical day?	Number of minutes, “do not know” or “prefer not to answer”

For each level of activity, the weekly duration was calculated by multiplying the number of days by the daily duration. This was then multiplied by the Metabolic Equivalent Task (MET) value to give the MET-minutes/week for an activity (MET values used: walking = 3.3, moderate-intensity = 4, vigorous-intensity = 8) [51,52]. MET is the ratio of energy expended per kilogram of body weight per hour to the standard resting metabolic rate (energy cost of a person at rest) [54]. It estimates the energy utilised and quantifies the intensity of physical activity [54]. For example, if a participant walks every day for 40 minutes, their walking MET-minutes/week is 924 (7×40×3.3).

The summed activity for each participant was calculated by adding the MET-minutes/week of the three levels of activity. For example, the summed activity of a person who walks every day for 40 minutes, does 30 minutes of moderate-intensity activity three days a week, and performs 40 minutes of vigorous-intensity activity three days a week is 2,244 MET-minutes/week (40×7×3.3 + 30×3×4 + 40×3×8).

Summed activity was also used to classify participants into IPAQ levels: low, moderate, and high [52]. The criteria for IPAQ levels are as follows [52]: high activity was defined as (a) vigorous-intensity activity on at least three days, achieving a minimum total physical activity of at least 1,500 MET-minutes/week, or (b) seven or more days of any combination of walking, moderate-intensity, or vigorous-intensity activities, achieving a minimum total physical activity of at least 3,000 MET-minutes/week. These are approximately equivalent to at least 60 minutes of moderate-intensity or vigorous-intensity activity each day. Moderate activity was defined as (a) three or more days of vigorous-intensity activity of at least 20 minutes per day, (b) five or more days of moderate-intensity activity and/or walking of at least 30 minutes per day, or (c) five or more days of any combination of walking, moderate-intensity, or vigorous-intensity activities, achieving a minimum total physical activity of at least 600 MET-minutes/week. These are equivalent to 30 minutes of moderate-intensity or vigorous-intensity activity on most days. Participants who did not meet the requirements for either group were placed in the low activity category.

Statistical analysis

Incident pancreatic cancer cases within the UK Biobank cohort were identified through linkage to national cancer registries (Health & Social Care Information Centre and the NHS Central Register) and coded using the 10th Revision of the International Classification of Diseases (ICD-10). Participants were followed up until PDAC diagnosis, loss to follow-up, final follow-up (October 31, 2015, for Scotland and March 31, 2016, for England and Wales), or death. The median (IQR) follow-up in this study was 6.8 (1.7) years.

Of the original 502,656 participants, 776 had pancreatic cancer at some point. Participants were excluded if their pancreatic cancer was not PDAC (due to their different pathology) (36) or if PDAC was diagnosed before enrolment (54). The number of PDAC cases was small compared to the total cohort, and the mean age (SD) of the PDAC cases was 61.6 (6.1) years, which was much higher than the mean (SD) age of the UK Biobank cohort, 56.5 (8.1). Therefore, a 1:4 nested case-control study was undertaken, matching for age (in five-year categories) and sex, with randomly selected controls (participants who did not have PDAC before or after enrolment). Participants were also excluded if they had incomplete data for physical activity or the variables that were adjusted (749). A total of 542 PDAC cases and 2,139 controls were used in the analyses.

Statistical tests were used to compare the baseline characteristics of PDAC cases and controls. The Pearson chi-squared test was used to investigate differences in the proportion of categorical variables. For continuous variables (all non-normally distributed), the Mann-Whitney U-test was used. Univariate and multivariate (adjusted) Cox proportional hazards models were used to estimate hazard ratios (HRs) and 95% confidence intervals (CIs) for the association between physical activity and PDAC. P-values of 0.05 or below were considered significant.

Walking, moderate-intensity, vigorous-intensity, and summed activities were each categorised into quartiles of total physical activity for analysis (first quartile = least activity). For each category of physical activity, the first quartile was the reference group, and the other quartiles were each compared to it. For the IPAQ levels, low activity was the reference group.

The multivariate models were adjusted for covariates and potential confounders identified from the literature and univariate analyses, including body mass index (BMI) (continuous), waist-to-hip ratio (WHR) (continuous), Townsend deprivation index (TDI) (continuous), diagnosed diabetes (yes, no), smoking status (never, previous, current), and alcohol intake frequency (never or special occasions, 1-3 times a month, 1-2 times a week, 3-4 times a week, daily or almost daily). Data were not available to adjust for chronic pancreatitis or family history of PDAC and pancreatitis. All analyses were conducted using IBM SPSS Statistics for Windows, Version 26 (Released 2019; IBM Corp., Armonk, New York).

## Results

Baseline characteristics

After a median (IQR) follow-up of 6.8 (1.7) years, 542 incident PDAC cases and 2,139 controls were identified and included in this study (n = 2,681). The baseline characteristics of the cases and controls are summarised in Table 2. Compared with the controls, the PDAC cases had higher proportions of diagnosed diabetes, current smokers, and higher BMI and waist-to-hip ratio. Summed weekly activity was lower in the cases than in the controls (summed activity quartiles and IPAQ activity levels).

**Table 2:** Baseline characteristics of PDAC cases and controls from the UK Biobank ^1^P-value not presented for matched variables (age and sex). ^2^Tested by Pearson chi-squared test for difference in the proportion of categorical variables. ^3^Tested by Mann-Whitney U-test for difference in continuous variables. *Quartile. PDAC: pancreatic ductal adenocarcinoma.

Variable	Controls (n=2 139), median (1^st^, 3^rd^ quartile), n (%)	PDAC (n=542), median (1^st^, 3^rd^ quartile), n (%)	P-value^1,2,3^
Age at enrolment (years)	63 (58, 66)	63 (58, 66)	Not applicable
Sex	-	-	Not applicable
Female	940 (43.9)	240 (44.3)	-
Male	1199 (56.1)	302 (55.7)	-
Diabetes	-	-	<0.01
No	1993 (93.2)	484 (89.3)	-
Yes	146 (6.8)	58 (10.7)	-
Smoking Status	-	-	<0.01
Never	1112 (52.0)	246 (45.4)	-
Previous	846 (39.6)	201 (37.1)	-
Current	181 (8.5)	95 (17.5)	-
Alcohol intake frequency	-	-	0.12
Never or special occasions	419 (19.6)	108 (19.9)	-
1-3 times a month	187 (8.7)	40 (7.4)	-
1-2 times a week	537 (25.1)	123 (22.7)	-
3-4 times a week	518 (24.2)	122 (22.5)	-
Daily or almost daily	478 (22.3)	149 (27.5)	-
Body mass index (kg/m^2^)	26.8 (24.5, 30.1)	27.4 (24.7, 30.6)	0.02
Waist/Hip ratio	0.9 (0.8, 1.0)	0.9 (0.8, 1.0)	0.04
Townsend deprivation index	-2.3 (-3.7, 0.2)	-2.1 (-3.6, 0.8)	0.11
Walking Metabolic Equivalent Task (MET)-minutes/week	-	-	0.32
Q*_1_ ≤330 (≤100)	563 (26.3)	159 (29.3)	-
Q_2_ 330-≤693 (100-≤210)	619 (28.9)	161 (29.7)	-
Q_3_ 693-≤1386 (210-≤420)	519 (24.3)	114 (21.0)	-
Q_4_ >1386 (>420)	438 (20.5)	108 (19.9)	-
Moderate-intensity activity MET-minutes/week (minutes per week)	-	-	0.77
Q_1_ ≤120, (≤30)	536 (25.1)	142 (26.2)	-
Q_2_ 120-≤480, (30-≤120)	560 (26.2)	133 (24.5)	-
Q_3_ 480-≤1440, (>120-≤360)	570 (26.6)	140 (25.8)	-
Q_4_ >1440, (>360)	473 (22.1)	127 (23.4)	-
Vigorous-intensity activity MET-minutes/week (minutes per week)	-	-	0.33
Q_1_ 0, (0)	920 (43.0)	243 (44.8)	-
Q_2_ ≤160, (≤20)	188 (8.8)	56 (10.3)	-
Q_3_ 160-≤720, (20-≤90)	507 (23.7)	128 (23.6)	-
Q_4_ >720, (>90)	524 (24.5)	115 (21.2)	-
Summed activity quartiles MET-minutes/week	-	-	<0.01
Q_1_ ≤828.3	514 (24.0)	156 (28.8)	-
Q_2_ 828.3-≤1836	541 (25.3)	135 (24.9)	-
Q_3_ 1836-≤3637	562 (26.3)	103 (19.0)	-
Q_4_ >3637	522 (24.4)	148 (27.3)	-
International Physical Activity Questionnaire (IPAQ) activity levels	-	-	0.04
Low	360 (16.8)	117 (21.6)	-
Moderate	907 (42.4)	217 (40.0)	-
High	872 (40.8)	208 (38.4)	-

Regression analysis

Evaluation of the physical activity variables and PDAC risk is shown in Table 3. Both univariate and multivariate analyses showed no significant associations in the walking, moderate-intensity or vigorous-intensity activity categories. In summed activity quartiles, only the third quartile had a significant reduction in PDAC risk in comparison to the first quartile (HR_univariate_ 0.62, 95% CI 0.49-0.80, p<0.01). This association remained significant after adjusting for potential confounders (aHR 0.67, 95% CI 0.52-0.86, p<0.01). In the univariate analysis, participants with moderate or high IPAQ levels showed a significant decrease in PDAC risk (HR_moderate_ 0.75, 95% CI 0.60-0.93, p<0.05 and HR_high_ 0.75, 95% CI 0.60-0.94, p<0.05). In the multivariate analysis, there was a borderline significant risk reduction of 20% with increased activity (aHR_moderate_ 0.80, 95% CI 0.63-1.00, p=0.05 and aHR_high_ 0.80, 95% CI 0.64-1.01, p=0.06).

**Table 3:** Risk of PDAC associated with physical activity (univariate and multivariable-adjusted analyses) ^1^Univariate analysis: matched for age and sex with no adjusting. ^2^Multivariate analysis: matched for age and sex and adjusted for: BMI (continuous), WHR (continuous), TDI (continuous), diagnosed diabetes (yes, no), smoking status (never, previous, current), alcohol intake frequency (never or special occasions, 1-3 times a month, 1-2 times a week, 3-4 times a week, daily or almost daily). *Quartile. PDAC: pancreatic ductal adenocarcinoma, BMI: body mass index, WHR: waist-to-hip ratio, TDI: Townsend deprivation index.

Physical activity variable	Hazard ratio^1 ^(95% CI)	P-value^1^	Hazard ratio^2^ (95% CI)	P-value^2^
Walking	-	-	-	-
Q*_1_ (reference group)	-	-	-	-
Q_2_	0.92 (0.74-1.15)	0.48	0.96 (0.77-1.20)	0.70
Q_3_	0.80 (0.63-1.01)	0.06	0.84 (0.66-1.07)	0.15
Q_4_	0.87 (0.68-1.11)	0.26	0.90 (0.71-1.16)	0.42
Moderate-intensity activity	-	-	-	-
Q_1_ (reference group)	-	-	-	-
Q_2_	0.89 (0.70-1.12)	0.31	0.90 (0.71-1.14)	0.39
Q_3_	0.93 (0.73-1.17)	0.52	0.95 (0.75-1.20)	0.64
Q_4_	0.99 (0.78-1.26)	0.93	1.02 (0.80-1.30)	0.88
Vigorous-intensity activity	-	-	-	-
Q_1_ (reference group)	-	-	-	-
Q_2_	1.14 (0.85-1.52)	0.39	1.18 (0.88-1.58)	0.27
Q_3_	0.95 (0.77-1.18)	0.65	1.00 (0.81-1.24)	0.99
Q_4_	0.85 (0.68-1.06)	0.14	0.88 (0.71-1.11)	0.28
Summed activity quartiles	-	-	-	-
Q_1_ (reference group)	-	-	-	-
Q_2_	0.83 (0.66-1.04)	0.11	0.86 (0.68-1.08)	0.19
Q_3_	0.62 (0.49-0.80)	<0.01	0.67 (0.52-0.86)	<0.01
Q_4_	0.94 (0.75-1.18)	0.60	0.97 (0.77-1.22)	0.81
IPAQ activity levels	-	-	-	-
Low (reference group)	-	-	-	-
Moderate	0.75 (0.60-0.93)	0.01	0.80 (0.63-1.00)	0.05
High	0.75 (0.60-0.94)	0.01	0.80 (0.64-1.01)	0.06

## Discussion

In the large UK Biobank cohort study, there was not strong evidence of an association between physical activity levels and the development of incident PDAC. No significant association was found for the different physical activity intensities: walking, moderate-intensity, or vigorous-intensity activities. Moderate and high IPAQ activity levels both showed a borderline significant 20% reduction in pancreatic cancer risk. Overall, there was no strong association between physical activity and pancreatic cancer.

Similar to this study, two prospective cohort studies also showed that different types of physical activity intensities did not reduce pancreatic cancer incidence [34,35]. However, a case-control study conducted in Minnesota [40] found that only light activity (OR 0.55, 95% CI 0.30-0.97) and moderate activity (OR 0.51, 95% CI 0.28-0.93) were associated with a reduced risk of pancreatic cancer, and not heavy activity (OR 1.18, 95% CI 0.66-2.11). In their study, participants reported the number of hours per week they usually spent on each of the activity levels during the year before pancreatic cancer diagnosis or in the past year for controls. This method has a high risk of recall bias, as participants were asked to recall information from far in the past, which can reduce the reliability of the results. In addition to the cohort studies, three systematic reviews that investigated the association between physical activity intensities and pancreatic cancer all concluded there was no association [43-45]. Along with the current literature, our study helps strengthen the evidence that different types of physical activity intensities do not reduce pancreatic cancer incidence.

The literature has inconsistent findings for the relationship between summed activity and pancreatic cancer incidence. Two prospective cohort studies and one case-control study found a significant reduction in pancreatic cancer cases with increased summed physical activity [28,37,40]. However, the findings of each study were specific to certain subgroups, which limits their generalisability. In a Finnish study on male smokers, it was found that combined occupational and leisure activity greater than sedentary levels reduced the risk of pancreatic cancer-for example, moderate/heavy activity in both settings (HR 0.42, 95% CI 0.22-0.83) [28]. The EPIC-Norfolk study showed that participants who had high activity and were younger than 60 years had reduced risks of pancreatic cancer (HR 0.27, 95% CI 0.07-0.99) [37]. It is of note that the study had only 88 cases in total, and only three cases were present in the high activity group of those younger than 60. Finally, a case-control study in Minnesota showed that only the second quartile (OR 0.51, 95% CI 0.30-0.85) and third quartile (OR 0.53, 95% CI 0.31-0.90) of total activity were associated with a reduced risk of pancreatic cancer, but not the highest quartile (OR 0.60, 95% CI 0.35-1.04) [40]. As stated previously, this study had limitations due to recall bias.

In contrast to these study findings, four prospective cohort studies concluded that summed activity levels were not associated with a decrease in pancreatic cancer incidence [31,33-35]. One of these was a large cohort study by Berrington de González et al. on the European Prospective Investigation into Cancer and Nutrition, which included 438,405 participants and had 324 incident cases of pancreatic cancer. They showed no significant reduction in pancreatic cancer risk when comparing the most and least active groups in their study (RR 0.82, 95% CI 0.50-1.35) [31].

Like the observational studies, the systematic reviews also had mixed findings. Two systematic reviews concluded no association [43,45], whereas one showed that higher summed activity reduced the risk of pancreatic cancer (five prospective studies, RR 0.72, 95% CI 0.52-0.99) [44]. The inconsistent results from the systematic reviews could be due to the use of different methodologies for categorising and recording physical activity.

In our study, summed activity showed a trend towards an inverse association (see Table 3). Of the summed activity quartiles, only the third quartile showed a significant reduction in pancreatic cancer incidence, and analysing IPAQ activity levels showed that increasing levels of activity were borderline significant. These results indicate that higher levels of summed activity may be protective against pancreatic cancer, but this association is weak, as shown by the statistical results. Furthermore, the non-significant results when comparing the most active quartile of summed activity with the least active, the high IPAQ activity group with the low activity group, and the non-significant findings for the different activity intensities (walking, moderate-intensity, and vigorous-intensity) suggest that there is no strong association between physical activity and pancreatic cancer incidence.

Strengths and limitations

This study was one of the largest single studies to investigate the impact of physical activity on pancreatic cancer incidence. Other major strengths include the use of a well-validated questionnaire and MET values to quantitatively analyse physical activity [51,52]. The questions assessed a range of physical activity domains [51,52], better representing the activity status of participants compared to studies where only one domain was analysed. Furthermore, a range of potential confounders were adjusted to increase the accuracy of the analysis.

This study also had some limitations. Repeat measures of physical activity over time were not undertaken in the UK Biobank Study. Therefore, physical activity measurements were not available from recruitment to the time of incident PDAC, making it impossible to undertake analyses with physical activity as a time-varying covariate in time-dependent regression models. Furthermore, physical activity was self-reported, introducing the possibility of response bias or misinterpretation. While there have been indications that the UK Biobank has a healthy volunteer selection bias, recent studies have shown that risk factor associations in the UK Biobank are generalisable [48,55,56]. To reduce inaccuracies due to participant reporting bias, accelerometers can be used to objectively analyse physical activity [57]. Currently, in the UK Biobank, there is insufficient data available to analyse the association between accelerometer data and pancreatic cancer incidence [57]. Future studies could use new technologies to increase the accuracy of physical activity recording. Additionally, the use of standardised definitions for the different intensities of physical activity would enable better analysis and comparisons between studies.

## Conclusions

Overall, this study does not show a strong association between physical activity and incident pancreatic cancer. No association was found between different physical activity intensities and pancreatic cancer. However, summed activity quartiles and IPAQ activity levels suggest that increasing summed physical activity may be protective.

## References

[1] Sung H, Ferlay J, Siegel RL, Laversanne M, Soerjomataram I, Jemal A, Bray F (2021). Global cancer statistics 2020: GLOBOCAN estimates of incidence and mortality worldwide for 36 cancers in 185 countries. CA Cancer J Clin.

[2] McGuigan A, Kelly P, Turkington RC, Jones C, Coleman HG, McCain RS (2018). Pancreatic cancer: a review of clinical diagnosis, epidemiology, treatment and outcomes. World J Gastroenterol.

[3] (2023). Pancreatic cancer statistics, Pancreatic Cancer UK. https://www.pancreaticcancer.org.uk/statistics/.

[4] Becker AE, Hernandez YG, Frucht H, Lucas AL (2014). Pancreatic ductal adenocarcinoma: risk factors, screening, and early detection. World J Gastroenterol.

[5] Ferlay J, Partensky C, Bray F (2016). More deaths from pancreatic cancer than breast cancer in the EU by 2017. Acta Oncol.

[6] (2023). Cancer Statistics Center, American Cancer Society. https://cancerstatisticscenter.cancer.org/#!/.

[7] (2023). ECIS - European Cancer Information System, European Commission. https://ecis.jrc.ec.europa.eu.

[8] Pourshams A, Sepanlou SG, Ikuta KS (2019). The global, regional, and national burden of pancreatic cancer and its attributable risk factors in 195 countries and territories, 1990-2017: a systematic analysis for the Global Burden of Disease Study 2017. Lancet Gastroenterol Hepatol.

[9] Singhi AD, Koay EJ, Chari ST, Maitra A (2019). Early detection of pancreatic cancer: opportunities and challenges. Gastroenterology.

[10] Moore SC, Lee IM, Weiderpass E (2016). Association of leisure-time physical activity with risk of 26 types of cancer in 1.44 million adults. JAMA Intern Med.

[11] McTiernan A, Friedenreich CM, Katzmarzyk PT (2019). Physical activity in cancer prevention and survival: a systematic review. Med Sci Sports Exerc.

[12] (2023). Physical activity and the risk of cancer, World Cancer Research Fund International. https://www.wcrf.org/diet-activity-and-cancer/risk-factors/physical-activity-and-cancer-risk/.

[13] Jacobs EJ, Chanock SJ, Fuchs CS (2010). Family history of cancer and risk of pancreatic cancer: a pooled analysis from the Pancreatic Cancer Cohort Consortium (PanScan). Int J Cancer.

[14] Bosetti C, Lucenteforte E, Silverman DT (2012). Cigarette smoking and pancreatic cancer: an analysis from the International Pancreatic Cancer Case-Control Consortium (Panc4). Ann Oncol.

[15] Fisher WE (2001). Diabetes: risk factor for the development of pancreatic cancer or manifestation of the disease?. World J Surg.

[16] Ben Q, Xu M, Ning X (2011). Diabetes mellitus and risk of pancreatic cancer: a meta-analysis of cohort studies. Eur J Cancer.

[17] Huxley R, Ansary-Moghaddam A, Berrington de González A, Barzi F, Woodward M (2005). Type-II diabetes and pancreatic cancer: a meta-analysis of 36 studies. Br J Cancer.

[18] Kirkegård J, Mortensen FV, Cronin-Fenton D (2017). Chronic pancreatitis and pancreatic cancer risk: a systematic review and meta-analysis. Am J Gastroenterol.

[19] Lo W, Morris MC, Ahmad SA, Patel SH (2019). Screening patients at high risk for pancreatic cancer - is it time for a paradigm shift?. J Surg Oncol.

[20] Bhosale P, Cox V, Faria S, Javadi S, Viswanathan C, Koay E, Tamm E (2018). Genetics of pancreatic cancer and implications for therapy. Abdom Radiol (NY).

[21] Storz P (2017). KRas, ROS and the initiation of pancreatic cancer. Small GTPases.

[22] (2023). Diet, nutrition, physical activity and pancreatic cancer, World Cancer Research Fund International. https://www.wcrf.org/diet-activity-and-cancer/cancer-types/pancreatic-cancer/.

[23] (2023). Physical activity and the risk of cancer, World Cancer Research Fund International. https://www.wcrf.org/diet-activity-and-cancer/risk-factors/physical-activity-and-cancer-risk/.

[24] Mann S, Beedie C, Balducci S, Zanuso S, Allgrove J, Bertiato F, Jimenez A (2014). Changes in insulin sensitivity in response to different modalities of exercise: a review of the evidence. Diabetes Metab Res Rev.

[25] McTiernan A (2008). Mechanisms linking physical activity with cancer. Nat Rev Cancer.

[26] Huang CJ, Zourdos MC, Jo E, Ormsbee MJ (2013). Influence of physical activity and nutrition on obesity-related immune function. ScientificWorldJournal.

[27] Michaud DS, Giovannucci E, Willett WC, Colditz GA, Stampfer MJ, Fuchs CS (2001). Physical activity, obesity, height, and the risk of pancreatic cancer. JAMA.

[28] Stolzenberg-Solomon RZ, Pietinen P, Taylor PR, Virtamo J, Albanes D (2002). A prospective study of medical conditions, anthropometry, physical activity, and pancreatic cancer in male smokers (Finland). Cancer Causes Control.

[29] Patel AV, Rodriguez C, Bernstein L, Chao A, Thun MJ, Calle EE (2005). Obesity, recreational physical activity, and risk of pancreatic cancer in a large U.S. Cohort. Cancer Epidemiol Biomarkers Prev.

[30] Sinner PJ, Schmitz KH, Anderson KE, Folsom AR (2005). Lack of association of physical activity and obesity with incident pancreatic cancer in elderly women. Cancer Epidemiol Biomarkers Prev.

[31] Berrington de González A, Spencer EA, Bueno-de-Mesquita HB (2006). Anthropometry, physical activity, and the risk of pancreatic cancer in the European prospective investigation into cancer and nutrition. Cancer Epidemiol Biomarkers Prev.

[32] Luo J, Iwasaki M, Inoue M, Sasazuki S, Otani T, Ye W, Tsugane S (2007). Body mass index, physical activity and the risk of pancreatic cancer in relation to smoking status and history of diabetes: a large-scale population-based cohort study in Japan--the JPHC study. Cancer Causes Control.

[33] Nöthlings U, Wilkens LR, Murphy SP, Hankin JH, Henderson BE, Kolonel LN (2007). Body mass index and physical activity as risk factors for pancreatic cancer: the multiethnic cohort study. Cancer Causes Control.

[34] Calton BA, Stolzenberg-Solomon RZ, Moore SC, Schatzkin A, Schairer C, Albanes D, Leitzmann MF (2008). A prospective study of physical activity and the risk of pancreatic cancer among women (United States). BMC Cancer.

[35] Stolzenberg-Solomon RZ, Adams K, Leitzmann M (2008). Adiposity, physical activity, and pancreatic cancer in the National Institutes of Health-AARP Diet and Health Cohort. Am J Epidemiol.

[36] Heinen MM, Verhage BA, Goldbohm RA, Lumey LH, van den Brandt PA (2011). Physical activity, energy restriction, and the risk of pancreatic cancer: a prospective study in the Netherlands. Am J Clin Nutr.

[37] Noor NM, Banim PJ, Luben RN, Khaw KT, Hart AR (2016). Investigating physical activity in the etiology of pancreatic cancer: the age at which this is measured is important and is independent of body mass index. Pancreas.

[38] Wu L, Zheng W, Xiang YB, Gao YT, Li HL, Cai H, Shu XO (2018). Physical activity and pancreatic cancer risk among urban Chinese: results from two prospective cohort studies. Cancer Epidemiol Biomarkers Prev.

[39] Hanley AJ, Johnson KC, Villeneuve PJ, Mao Y (2001). Physical activity, anthropometric factors and risk of pancreatic cancer: results from the Canadian enhanced cancer surveillance system. Int J Cancer.

[40] Zhang J, Dhakal IB, Gross MD, Lang NP, Kadlubar FF, Harnack LJ, Anderson KE (2009). Physical activity, diet, and pancreatic cancer: a population-based, case-control study in Minnesota. Nutr Cancer.

[41] Brenner DR, Wozniak MB, Feyt C (2014). Physical activity and risk of pancreatic cancer in a central European multicenter case-control study. Cancer Causes Control.

[42] Kollarova H, Azeem K, Tomaskova H (2014). Is physical activity a protective factor against pancreatic cancer?. Bratisl Lek Listy.

[43] Bao Y, Michaud DS (2008). Physical activity and pancreatic cancer risk: a systematic review. Cancer Epidemiol Biomarkers Prev.

[44] O'Rorke MA, Cantwell MM, Cardwell CR, Mulholland HG, Murray LJ (2010). Can physical activity modulate pancreatic cancer risk? A systematic review and meta-analysis. Int J Cancer.

[45] Behrens G, Jochem C, Schmid D, Keimling M, Ricci C, Leitzmann MF (2015). Physical activity and risk of pancreatic cancer: a systematic review and meta-analysis. Eur J Epidemiol.

[46] Farris MS, Mosli MH, McFadden AA, Friedenreich CM, Brenner DR (2015). The association between leisure time physical activity and pancreatic cancer risk in adults: a systematic review and meta-analysis. Cancer Epidemiol Biomarkers Prev.

[47] Allen N, Sudlow C, Downey P (2012). UK Biobank: current status and what it means for epidemiology. Health Policy Technol.

[48] (2023). UK Biobank: protocol for a large-scale prospective epidemiological resource. United Kingdom (UK) Biobank. https://www.ukbiobank.ac.uk/media/gnkeyh2q/study-rationale.pdf.

[49] The Ethics and Governance Council (2023). The Ethics and Governance Council, United Kingdom (UK) Biobank.. https://www.ukbiobank.ac.uk/the-ethics-and-governance-council/.

[50] Townsend P (1987). Deprivation. J Soc Policy.

[51] Craig CL, Marshall AL, Sjöström M (2003). International physical activity questionnaire: 12-country reliability and validity. Med Sci Sports Exerc.

[52] (2023). Score the iPAQ. International physical activity questionnaire (iPAQ). https://sites.google.com/view/ipaq/score.

[53] (2023). Category 100054. United Kingdom (UK) Biobank. https://biobank.ctsu.ox.ac.uk/crystal/label.cgi?id=100054.

[54] Ainsworth BE, Haskell WL, Herrmann SD (2011). 2011 Compendium of Physical Activities: a second update of codes and MET values. Med Sci Sports Exerc.

[55] Batty GD, Gale CR, Kivimäki M, Deary IJ, Bell S (2020). Comparison of risk factor associations in UK Biobank against representative, general population based studies with conventional response rates: prospective cohort study and individual participant meta-analysis. BMJ.

[56] Fry A, Littlejohns TJ, Sudlow C (2017). Comparison of sociodemographic and health-related characteristics of UK Biobank participants with those of the general population. Am J Epidemiol.

[57] Doherty A, Jackson D, Hammerla N (2017). Large scale population assessment of physical activity using wrist worn accelerometers: the UK Biobank study. PLoS One.

